# Impact of socioeconomic status on the clinical outcomes in hospitalised patients with SARS-CoV-2 infection: a retrospective analysis

**DOI:** 10.1007/s10389-022-01730-2

**Published:** 2022-07-06

**Authors:** Lucio Boglione, Valentina Dodaro

**Affiliations:** 1 Department of Translational Medicine (DiMET), University of Eastern Piedmont, Via Solaroli 17, 28100 Novara, Italy; 2 Department of Medical Sciences, University of Turin, Turin, Italy

**Keywords:** SARS-CoV-2, COVID-19, socioeconomic status, health inequalities, educational level

## Abstract

**Aim:**

A disadvantaged socioeconomic status (SES) was previously associated with higher incidence and poor outcomes both of non-communicable diseases (NCDs) and infectious diseases. Inequalities in health services also have a negative effect on the coronavirus disease 2019 (COVID-19) morbidity and mortality.

**Subject and methods:**

The study analysed the role of SES measured by the educational level (EL) in hospitalised patients with COVID-19 between 9 March 2020 and 20 September 2021 at our centre of infectious diseases. Clinical outcomes were: length of hospitalisation, in-hospital mortality and the need of intensive-care-unit (ICU) support.

**Results:**

There were 566 patients included in this retrospective analysis. Baseline EL was: illiterate (5, 0.9%), primary school (99, 17.5%), secondary school (228, 40.3%), high school (211, 37.3%), degree (23, 4.1%); median age was higher in low EL (72.5 years vs 61 years, *p* = 0.003), comorbidity (56% in low EL, 34.6% in high EL, *p* < 0.001), time from the symptoms and PCR diagnosis (8.5 days in low EL, 6.5 days in high EL, *p* < 0.001), hospitalisation length (11.5 days in low EL, 9.5 days in high EL, *p* = 0.011), mortality rate (24.7% in low EL, 13.2% in high EL, *p* < 0.001). In the multivariate analysis there were predictors of mortality: age (OR = 4.981; 95%CI 2.172–11.427; *p* < 0.001), comorbidities (OR = 3.227; 95%CI 2.515–11.919; *p* = 0.007), ICU admission (OR = 6.997; 95%CI 2.334–31.404; *p* = 0.011), high vs low EL (OR = 0.761; 95%CI 0.213–0.990; *p* = 0.021). In survival analysis, higher EL was associated with a decreased risk of mortality up to 23.9%.

**Conclusion:**

Even though the EL is mainly related to the age of patients, in our analysis, it resulted as an independent predictor of in-hospital mortality and hospitalisation time. Unfortunately, this is a study focused only on hospitalised patients, and we did not examine the possible effect of EL in outpatients. Further analyses are required to confirm this suggestion and provide novel information.

## Introduction

The pandemic, due to severe acute respiratory syndrome coronavirus 2 (SARS-CoV-2), rapidly became a global health problem, with important clinical, social and epidemiological consequences caused by this new coronavirus disease 2019 (COVID-19) (Guan et al. 2020; De Rosa et al. 2021). Several major risk factors were associated with mortality, such as age, obesity, comorbidities and gender (De Rosa et al. 2021); the role of socioeconomic status (SES) was recently observed as determinant in COVID-19 hospital admissions and mortality (Bambra et al. 2020; Patel et al. 2020; Little et al. 2021) as previously reported for other important respiratory infection (Hawker et al. 2003; Rutter et al. 2012; Khalatbari-Soltani et al. 2020). Unfortunately, the assessment of SES was not routinely examined in most studies about COVID-19, and this aspect may be underestimated as a determinant of the risk of infection, hospitalisation and poor outcomes in SARS-CoV-2 infection (Greenaway et al. 2020; Marmot and Allen 2020). Disadvantaged SES was related to poor living and working conditions, lower-paid work (especially in the basic services) with limited healthcare services and level of education (Galobardes et al. 2007). Furthermore, some comorbidities such as diabetes or cardiovascular diseases were more frequent in patients with poor SES, with a consequent higher risk of hospitalisation and mortality (Galobardes et al. 2006). In these conditions the poverty status possibly lead to a reduced compliance for social distancing, with an increased risk of virus exposure and a reduction in the immune system’s effectiveness (Cookson et al. 2009). As for non-communicable chronic diseases (NCDs), people with disadvantaged SES were admitted to hospital with advanced stage of illness, which were more severe or critical and consequently there was a higher risk of mortality (Szczepura 2005).

The aim of this retrospective study was the analysis of the SES impact on the clinical outcomes in a group of hospitalised patients affected by COVID-19.

## Methods

### Study design and definition

This is a single-centre, observational, retrospective study considering all the consecutive patients hospitalised at our Infectious Diseases unit of St. Andrea Hospital, Vercelli, Italy, between 9 March 2020 and 20 September 2021, with a confirmed diagnosis of SARS-CoV-2 infection based on a positive result on a real-time reverse transcriptase-polymerase-chain reaction (RT-PCR) of a nasal or pharyngeal swab specimen; relevant demographic, clinical and therapeutic data were recorded. Age, sex, body mass index (BMI), provenance (home, long-term care, hospital), comorbidities and laboratory baseline examinations were reported. Comorbidities included all chronic diseases: coronary artery disease (CAD), chronic obstructive pulmonary disease (COPD), diabetes, hypertension, neurological, psychiatric, kidney, neoplastic and immunological diseases. Defined clinical outcomes were: death, intensive-care-unit (ICU) admission, need of non-invasive ventilation (NIV), occurrence of sepsis. Occupational status was defined as: employed, unemployed and retired. Individual SES was estimated using the educational level (EL) according to different schooling conditions: illiterate, primary school, secondary school, high school or degree. High EL was defined as high school or graduate, low EL as illiterate, primary and secondary school.

### Study endpoints

The primary endpoint was the comparison of mortality between patients with high and low EL; secondary endpoints were the assessment of length of hospitalisation, need of NIV or ICU admission between the two groups of patients.

### Statistical analysis

In descriptive statistics, continuous variables were summarised as median (inter-quartile range (IQR): 25th to 75th percentiles). Categorical variables were described as frequency and percentage. All data were assessed for normality using a Shapiro–Wilk test and categorical data were compared using a Mann–Whitney or Kruskal–Wallis statistical test. To investigate continuous data, a Spearman rank correlation was utilised. The association was calculated using the χ^2^-test. Multivariate logistic regression analysis with a stepwise forward selection was performed for mortality evaluation with p-values of less than 0.05 as the criteria for model inclusion. All *p*-values were two-tailed. *P* <0.05 was considered statistically significant. Survival analysis was carried out comparing the two groups using the Kaplan–Meier plot and compared with the log-rank (Mantel–Cox) test. Statistical analyses were conducted using SPSS software package ver. 26.0 (Chicago, IL, USA).

## Results

### Patient selection and baseline characteristics

Included in the study period were 696 patients who had been hospitalised with a confirmed diagnosis of SARS-CoV-2 infection; 130 patients were further excluded from this analysis due to the lack of schooling data in the clinical documentation; in the end, 566 patients were included in this retrospective analysis. Baseline characteristics of the study population were reported in Table 1. Median age was 68 years; 362 (63.9%) patients were male; median BMI was 24.5; the different provenance were: long-term care facilities (144, 25.4%), hospital (71, 12.5%) and home (351, 62%). Education level was: illiterate (5, 0.9%), primary school (99, 17.5%), secondary school (228, 40.3%), high school (211, 37.3%) and degree (23, 4.1%). Occupational status was: employed (329, 58.1%), unemployed (54, 9.5%) and retired (183, 32.3%). The most prevalent comorbidities were: CAD (102, 37.6%), diabetes (103, 18.2%), hypertension (195, 34.4%), neurological disease (57, 10.1%), psychiatric disease (31, 5.5%), immunological disease (25, 4.4%), COPD (42, 7.2%), kidney disease (13, 2.3%) and malignancies (18, 3.2%). Median time from the symptoms’ onset to PCR diagnosis was 7.5 days; interstitial pneumonia was diagnosed in 428 patients (75.6%); 246 (43.5%) patients were given NIV/CPAP, and 88 (15.2%) required further ICU admission. Median hospitalisation length was 12.5 days; antiviral treatment was given in 181 patients (31.9%), corticosteroids in 359 (63.4%); overall mortality rate was 19.9% and sepsis occurred in 62 patients (11%).

**Table 1:** Baseline characteristics of the study population

Characteristics	Overall patients (n = 566)
Age (median, IQR)	68 [57–78]
Male sex (n, %)	362 (63.9)
BMI (median, IQR)	24.5 [22.5–27.5]
Provenance (n, %)	
–long-term care	144 (25.4)
–hospital	71 (12.5)
–home	351 (62)
Education level:	
–illiterate	5 (0.9)
–primary school	99 (17.5)
–secondary school	228 (40.3)
–high school	211 (37.3)
–degree	23 (4.1)
Occupational status:	
–employed	329 (58.1)
–unemployed	54 (9.5)
–retired	183 (32.3)
Comorbidity (n, %)	
–CAD (coronary artery disease)	102 (37.6)
–diabetes	103 (18.2)
–hypertension	195 (34.4)
–neurological disease	57 (10.1)
–psychiatric disease	31 (5.5)
–immunological disease	25 (4.4)
–COPD	41 (7.2)
–kidney disease	13 (2.3)
–malignancies	18 (3.2)
Days from the symptoms to PCR diagnosis (median, IQR)	7.5 [6–11.5]
Interstitial pneumonia (n, %)	428 (75.6)
NIV/CPAP (n, %)	246 (43.5)
ICU admission (n, %)	88 (15.2)
WBC (10^9^/L)	6220 [3405–9844]
Platelets (10^9^/L)	188 [105–334]
eGFR (mL/min)	61 [40–105]
CRP (mg/L)	10.5 [4.2–14.8]
Ferritin (ng/mL)	713 [338–1718]
D-dimer (ng/mL)	430 [237–900]
P/F (median, IQR)	265 [211–310]
Days of hospitalization (median, IQR)	12.5 [6.5–14.5]
Antiviral treatment (n, %)	181 (31.9)
Corticosteroid treatment (n, %)	359 (63.4)
Sepsis (n, %)	62 (11)
Death (n, %)	113 (19.9)

In Table 2, the baseline characteristics were reported with a significant difference distribution according to EL; median age was higher in low EL (72.5 years vs 61 years, *p* = 0.003), comorbidity (56% in low EL, 34.6% in high EL, *p* < 0.001), time from the symptoms and PCR diagnosis (8.5 days in low EL, 6.5 days in high EL, *p* < 0.001), hospitalisation length (11.5 days in low EL, 9.5 days in high EL, *p* = 0.011), mortality rate (24.7% in low EL, 13.2% in high EL, *p* < 0.001).

**Table 2:** Different baseline characteristics and mortality in the study population by patients’ educational level

	High EL (n = 234)	Low EL (n = 332)	*P* value
Age (median, IQR)	61 [55–75]	72.5 [68.5–85.5]	0.003
Comorbidity (n, %)	81 (34.6)	189 (56.9)	<0.001
Days from the symptoms to PCR diagnosis (median, IQR)	6.5 [5.2–9]	8.5 [7.5–12.5]	<0.001
Days of hospitalization (median, IQR)	9.5 [7.5–10]	11.5 [8–14.5]	0.011
Death (n, %)	31 (13.2)	82 (24.7)	<0.001

### Univariate and multivariate analysis considering the mortality in the study population

In univariate analysis age, sex, comorbidities, BMI, NIV, ICU admission, high vs low EL, antiviral and corticosteroid therapy were considered for mortality; the factors found to be significantly associated with mortality were: age (OR = 4.112; 95%CI 1.448–15.009; *p* < 0.001), NIV (OR = 3.821; 95%CI 1.844–7.992; *p* = 0.016); ICU admission (OR = 5.692; 95%CI 2.661–21.679; *p* < 0.001), high vs low EL (OR = 0.680; 95%CI 0.451–0.975; *p* = 0.025) and comorbidities (OR = 3.561; 95%CI 2.926–12.765; *p* = 0.004). In multivariate analysis the predictors of mortality were: age (OR = 4.981; 95%CI 2.172–11.427; *p* < 0.001), comorbidities (OR = 3.227; 95%CI 2.515–11.919; *p* = 0.007), ICU admission (OR = 6.997; 95%CI 2.334–31.404; *p* = 0.011) and high vs low EL (OR = 0.761; 95%CI 0.213–0.990; *p* = 0.021) (Table 3).

**Table 3:** Univariate and multivariate logistic regression considering the mortality in the study population

Univariate analysis	
**Factors**	**OR, 95%CI,** ***p***
**Age**	4.112 (1.448–15.009) ***p*** < 0.001
Sex	1.191 (0.556–8.174) *p* = 0.629
BMI	2.343 (0.917–4.065) *p* = 0.881
**Comorbidities**	3.561 (2.926–12.765) ***p*** = 0.004
**NIV**	3.821 (1.844–7.992) ***p*** = 0.016
**ICU admission**	5.692 (2.661–21.679) ***p*** < 0.001
**High EL vs low EL**	0.680 (0.451–0.975) ***p*** = 0.025
Antiviral therapy	0.772 (0.431–2.571) *p* = 0.422
Corticosteroid therapy	0.667 (0.340–13.991) *p* = 0.775
**Multivariate analysis**	
**Factors**	**OR, 95% CI,** ***p***
**Age**	4.981 (2.172–11.427) ***p*** < 0.001
**Comorbidities**	3.227 (2.515–11.919) ***p*** = 0.007
NIV	2.812 (0.981–5.636) *p* = 0.081
**ICU admission**	6.997 (2.334–31.404) ***p*** = 0.011
**High EL vs low EL**	0.761 (0.213–0.990) ***p*** = 0.021

### Survival analysis

**Fig. 1:**
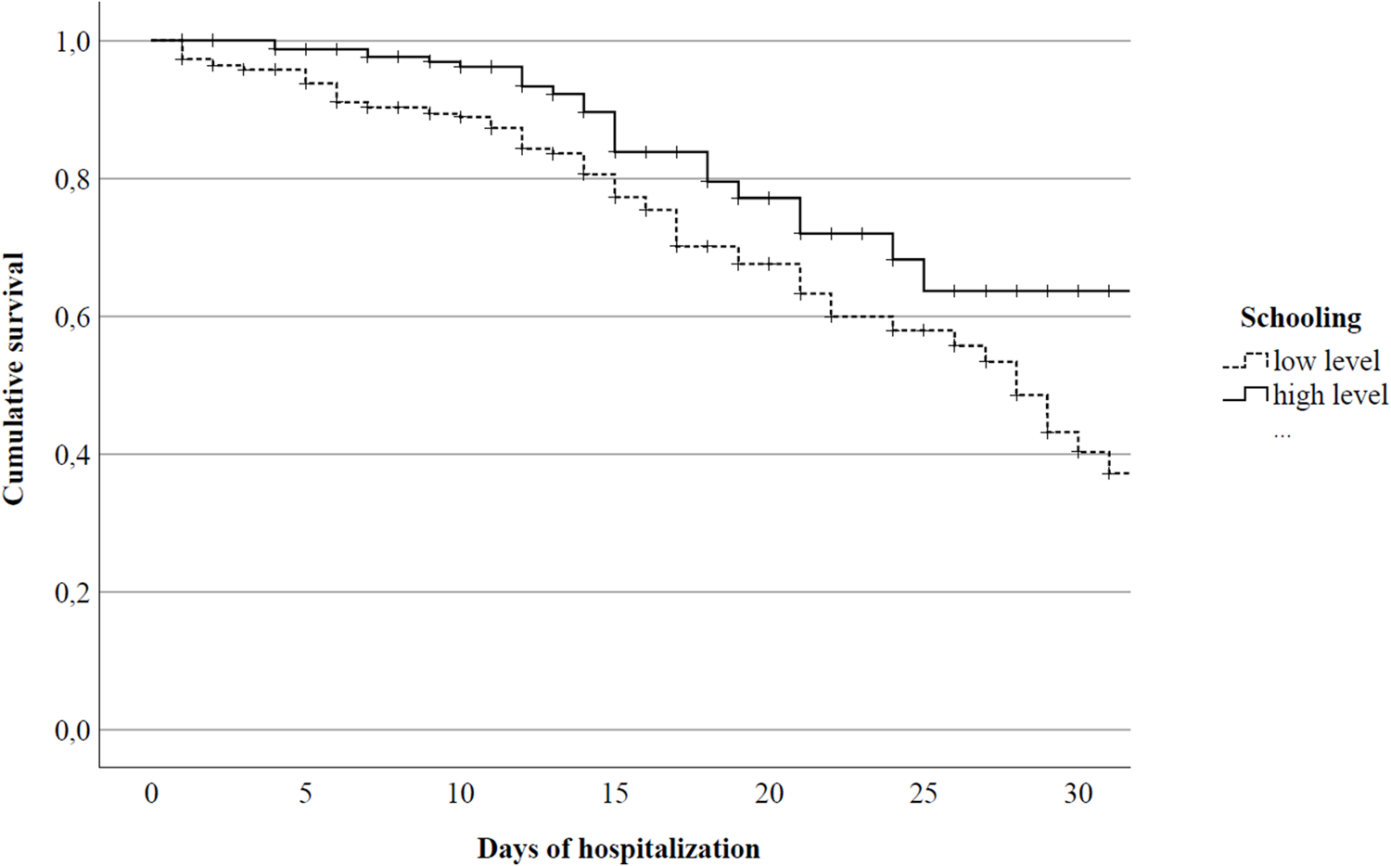
Survival analysis in hospitalised patients with COVID-19 according to educational level

Survival analysis was carried out comparing the patients with high vs low EL (Fig. 1) with a significant difference between the two groups (χ^2^ = 10.170, *p* < 0.001). Higher EL was associated with a decreased risk of mortality compared to lower EL by up to 23.9%.

## Discussion

Evidence of significant health inequalities were historically documented from the 1918 influenza pandemic (Sydenstricker 2006) and were related to the different impacts of social determinants in the affected population. The mortality rate was higher in countries with elevated pre-existing social and economic inequalities, with the concurrent effect of viral infections and underlying NCDs. SES measurement can be done through several indicators such as social position, geographical residence, job or educational level. The role of EL was previously analysed in health inequalities (Mackenbach and Kunst 1997): the schooling level represents the availability of cultural resources, and the relationship with health may be directly conditioned through cognitive functions such as the ability to understand news, communications and the rules of conduct in both preventive or therapeutic practice; on the other hand, it is indirectly related to SES not only of the single patient but also of the whole family and strongly influences the future employment and income (Davey Smith et al. 1998). The main advantage of EL is the availability of clinical and demographic data, and stability over time regardless of employment or income. The main limitations are the dependence on age and the significance of EL in the different birth groups, the measurement (continuous, with number of years or categorial, with different levels) and the lack of quality assessment (Yen and Moss 1999).

**Fig. 2:**
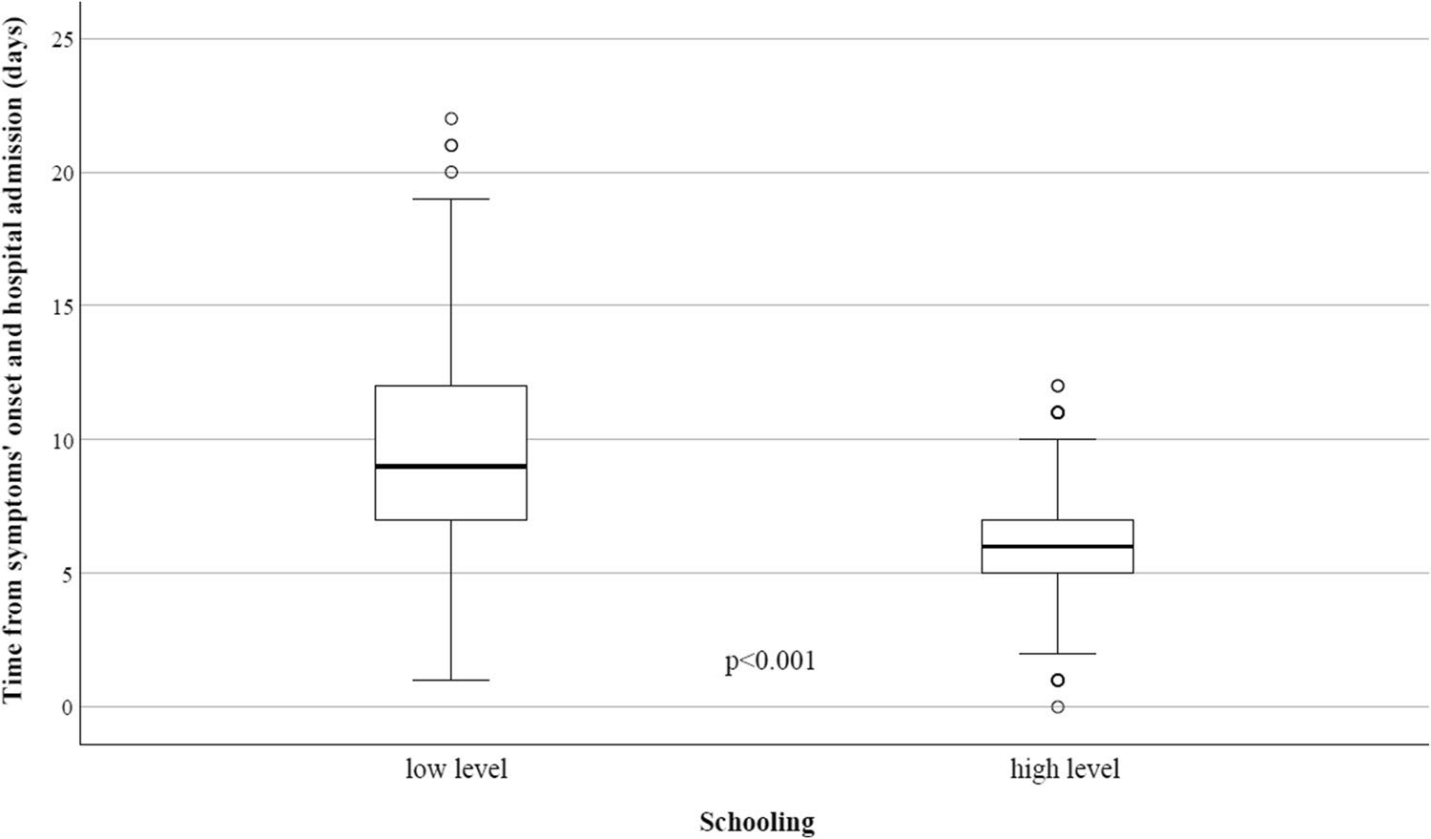
Time from symptoms’ onset and hospital admission according to educational level

In our study we report the analysis of EL on clinical outcomes (mortality and length of hospitalisation) in a group of hospitalised patients with COVID-19. As expected, and previously reported, the age of enrolled patients and the need of ICU admission were found as main factors related to in-hospital mortality. This is not surprising because older patients were more frequently affected by one or more comorbidities and were at higher risk of illness progression with an unfavourable outcome. In our group we evidenced that the EL was related to age (71 vs 61 years in low and high level, respectively) and comorbidities (56% vs 34% in low and high level, respectively); this aspect consequently led to higher mortality and a longer time of hospitalisation in patients with lower EL. However, despite the effect of age, comorbidities and other clinical characteristics on mortality, we confirmed a direct impact of the SES measured by EL independently from the other variables. Low EL was independently associated with higher risk of mortality compared to higher EL (adjusted OR = 0.761; 95%CI 0.213–0.990). Although this value is strongly related to the local characteristics of the study population, such as age, comorbidities, health management and protocols, we found a similar effect of SES evaluation as reported in other studies (Franchi and Gili 2020; Little et al. 2021; Politi et al. 2021), and this aspect is therefore encouraging as it confirms the validity of this data. Another interesting finding is the time lapse between the onset of symptoms and hospital admission (Fig. 2): it is now well known that most clinical and therapeutical approaches to COVID-19 required an early diagnosis as a delayed diagnosis maybe led to frequent clinical complications, longer hospitalisation, and higher mortality, especially in older patients with comorbidities. The reasons why the EL caused this delay were not clear; however, we can make some assumptions: a first hypothesis is that in the initial phase of the pandemic with the major restrictions due to strict lockdown measures, the most disadvantaged patients tried to avoid the hospital admission owing to the healthcare overload and the fear of ‘unknown aspects’ of this illness amplified by the news, media and unverified information. A second assumption is that the lower EL limits the availability of healthcare services and primarily the correct home management of the first symptoms and drugs intake: for example, some patients reported taking medication found to be inappropriate in the indication, dose and timing, or they were not capable of self-monitoring when clinical conditions have worsened.

The unfavourable outcomes observed in the disadvantaged population were mainly related to a higher prevalence of comorbidities such as cardiovascular diseases, chronic obstructive pulmonary disease, diabetes and hypertension (Lantz et al. 2001); these conditions were associated with higher mortality, a longer time of hospitalisation with consequently major risks of other clinical complications such as hospital infections and the need of ICU admission.

**Fig. 3:**
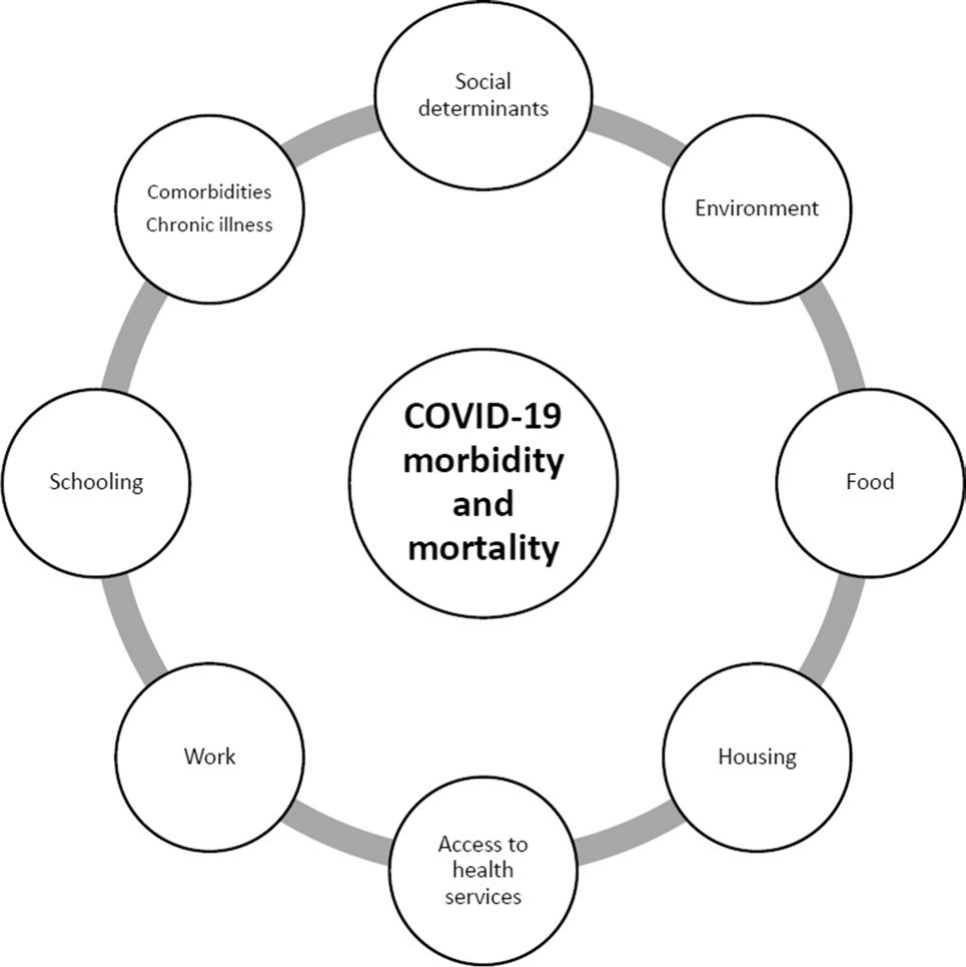
Syndemic of clinical and non-clinical conditions with COVID-19 morbidity and mortality

The COVID-19 pandemic overlaps with other underlying factors: non-communicable diseases, environmental and geographical conditions, social and educational determinants, access to services and work, resulting in a ‘syndemic’ enhancement of severity and spread in SES disadvantages (Fig. 3).

Finally, this study suggests that the SES measured by EL is an independent predictor of clinical outcomes during COVID-19 hospitalisation. This element should serve to focus the attention on the role of social determinants in health management during the COVID-19 pandemic, and more broadly on the inequalities in health policies.

The present study was focused only on hospitalised patients and mainly analyses the impact of EL on mortality in a limited setting of a population infected by SARS-CoV-2; it is therefore possible that this effect is emphasised by a limited selection of patients characterised by a major frailty and risk of poor outcomes. Moreover, we have not studied the real incidence of SARS-CoV-2 infection in non-hospitalised or asymptomatic patients, and we are unable to confirm whether the EL can also affect the greater susceptibility to viral infection.

## Data Availability

The reported data underlying this article will be shared on reasonable request to the corresponding author.
